# Preoperative esophageal cancer staging assessment based on intravoxel incoherent motion and apparent diffusion coefficient: a comparative study of maximum-diameter slice region of interest and whole volume of interest analysis

**DOI:** 10.1186/s12880-025-01973-x

**Published:** 2025-11-04

**Authors:** Feng Feng, Lin Kong, Chen Yang, Yong Wang, Jin Chen, Jianwen Zhou, Yifan Hu

**Affiliations:** 1 Department of Radiology, Affiliated Tumor Hospital of Nantong University, Nantong, Jiangsu Province China; 2 Department of Radiology, Dongtai People’s Hospital, Medical School of Nantong University, 2# Kangfuxi Rd, Dongtai, Yancheng, Jiangsu Province 224200 China

**Keywords:** ESCC, IVIM, ADC, T staging, N staging

## Abstract

**Purpose:**

MRI-based intravoxel incoherent motion (IVIM) and apparent diffusion coefficient (ADC) parameters evaluate molecular diffusion and microvascular perfusion. This study assessed their utility in esophageal squamous cell carcinoma (ESCC) staging, compared different measurement approaches, and explored their predictive value for surgical staging.

**Methods:**

Eighty prospectively enrolled ESCC patients (61 males, 19 females; median age 70 years) from February 2020 to August 2020 underwent 3.0T IVIM-DWI with respiratory-triggered and fat saturation techniques. Tumor ADC and IVIM parameters were calculated (b-values: 0–1000 s/mm²) for maximum-diameter slices and whole-volume regions of interest (ROI), and their correlations with T (tumor)/N (lymph node involvement) staging were analyzed.

**Results:**

Full-volume measurements demonstrated higher interobserver reproducibility than single-slice measurements. For T staging, tumors with lower single-slice ADC_max and D_min values, as well as lower whole-volume ADC_std, were more likely to present with advanced stages (all *p* < 0.05). Parameters derived from the maximum-diameter slice provided higher diagnostic accuracy for T-stage discrimination compared with whole-volume analysis (AUC 0.75 vs. 0.70). For N staging, single-slice D_mean was associated with nodal involvement, while whole-volume analysis revealed that lower D_max, f_mean, f_min, and D*_std along with higher D*_min were correlated with lymph node metastasis, yielding better diagnostic performance (AUC 0.70 vs. 0.60).

**Conclusion:**

MRI-derived IVIM and ADC parameters provide non-invasive biomarkers for ESCC staging with the potential to guide preoperative decision-making, while exploring different ROI delineation strategies may further enhance research and clinical application.

**Supplementary Information:**

The online version contains supplementary material available at 10.1186/s12880-025-01973-x.

## Introduction

Esophageal cancer (EC) is a leading malignancy, ranking seventh in cancer-related mortality [1]. Accurate preoperative staging is essential for treatment planning, as different stages require distinct therapeutic approaches [2]. According to the 2023 NCCN Guidelines, T3-T4a tumors penetrating the esophageal adventitia typically require neoadjuvant chemoradiotherapy followed by surgery, whereas T1-T2 tumors without lymph node metastasis are primarily treated with surgery alone [3].

Preoperative EC evaluation mainly includes multidetector computed tomography (MDCT), endoscopic ultrasound (EUS), magnetic resonance imaging (MRI), and positron emission tomography/computed tomography (PET/CT). EUS is the standard for preoperative staging but is invasive and challenging in large tumors [4, 5]. CT and PET/CT have limited soft tissue resolution, affecting staging accuracy [6, 7].

MRI, recognized for its superior soft tissue resolution, can accurately depict the eight histopathological layers of the esophagus and evaluate the extent of tumor involvement in vitro studies [8]. Advances in MRI technology have led to its increasing application in the clinical staging of EC [6, 9]. However, conventional MRI sequences, such as T1-weighted images (T1WI) and T2-weighted images (T2WI), remain limited in reliably determining tumor involvement of the thin esophageal adventitia [10].

Functional imaging techniques have opened new avenues for the preoperative staging of EC. Diffusion-weighted imaging (DWI) has emerged as a valuable tool for tumor detection, characterization, and treatment response monitoring [11, 12]. Nevertheless, the apparent diffusion coefficient (ADC) derived from traditional DWI is unable to effectively differentiate between diffusion and perfusion effects. Intravoxel incoherent motion (IVIM) imaging, which quantifies diffusion (D), pseudo-diffusion (D*), and perfusion fraction (f), allows simultaneous assessment of molecular diffusion and microvascular perfusion. Compared to conventional ADC, IVIM has demonstrated greater utility in tumor evaluation and treatment monitoring [13, 14].More importantly, these functional parameters may provide information that directly complements anatomical imaging. For T-staging, diffusion and perfusion metrics can reflect tumor cellularity and angiogenesis, which are associated with deeper mural invasion beyond the esophageal adventitia. For N-staging, conventional size- and morphology-based criteria for lymph nodes are often insufficient, whereas the diffuse perfusion characteristics may help determine whether there is lymph node metastasis [15].

Despite these advantages, most IVIM studies rely on measurements from a single slice or a few selected maximum slices, raising concerns about their ability to represent the tumor’s overall characteristics and reproducibility. Therefore, this study aimed to evaluate the clinical utility of IVIM-derived functional parameters compared with conventional ADC for preoperative T and N staging of esophageal squamous cell carcinoma (ESCC), and to determine whether whole-volume measurements offer superior accuracy and reproducibility over single-slice approaches. We hypothesized that IVIM, particularly with whole-volume analysis, would outperform conventional ADC and single-slice approaches in staging performance.

## Materials and methods

### Study population

**Fig. 1:**
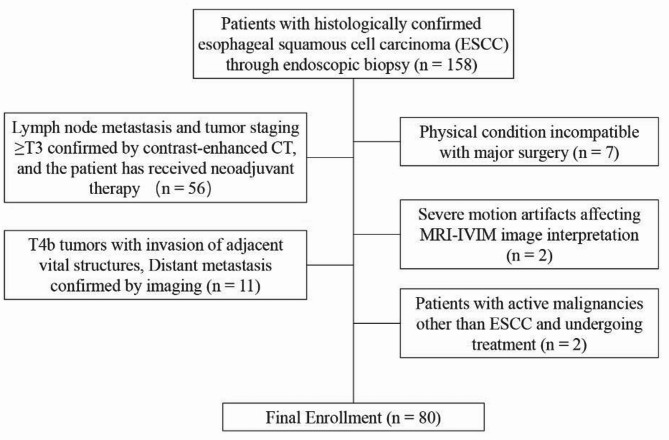
Enrollment flow chart

This study was approved by the Ethics Committee of Dongtai People’s Hospital (No.: 2020-dtry-K-16) and conducted in accordance with the Declaration of Helsinki. Written informed consent was obtained from all subjects. Between January 2020 and September 2022, 158 consecutive patients with endoscopically confirmed esophageal squamous cell carcinoma (ESCC) who met the following criteria were initially enrolled: (1) histopathological diagnosis of ESCC; (2) scheduled for surgical resection with preoperative IVIM-DWI MRI performed within 2 weeks prior to surgery. Following rigorous screening, 78 patients were excluded (Fig. 1), resulting in 80 eligible participants undergoing both MRI evaluation and surgical intervention.

### MRI protocol

MRI was conducted using a 3.0T scanner (Discovery MR 750 W, GE Medical Systems, Waukesha, WI, USA) with a 32-channel body coil. Patients fasted for 6 h and practiced shallow breathing. IVIM-DWI imaging was performed with localized shimming over the chest region, including the esophagus, respiratory-triggered acquisition, and fat suppression using chemical shift selective saturation to minimize artifacts. The IVIM-DWI parameters included TR ≈ 3000ms, TE minimum, slice thickness 4.4 mm, inter-slice gap 1.0 mm, FOV 42 × 42 cm, and matrix size 128 × 128. Eleven b-values (0, 20, 50, 80, 100, 150, 200, 400, 600, 800, 1000s/mm2) were used, with corresponding excitations of 2, 2, 2, 2, 2, 2, 2, 3, 3, 4, and 6, respectively. The total IVIM-DWI acquisition time was approximately 6 min. Additional sequences included T1WI, T2WI, and fat-suppressed T2WI.

### Imaging analysis

All images were acquired and processed using Image Engine software (Vusion Tech) [16]. IVIM data were independently evaluated by two radiologists (9 and 15 years of experience) blinded to histopathological results. ADC values were calculated using a mono-exponential model with 11 b-values, based on the formula:

$$\:S\left(b\right)={S}_{0}\cdot\:{e}^{-b\cdot\:ADC}$$

where S(b) is the signal intensity at a given b-value, 𝑆_0_ is the signal intensity without diffusion weighting (b = 0).

For the true ADC (D, also referred to as ADC_slow_), pseudo ADC (D∗, also referred to as ADC_fast_), and perfusion fraction (*f*), a bi-exponential IVIM model was applied, according to the equation:

$$\:S\left(b\right)={S}_{0}\cdot\left[\right(1-f)\cdot{e}^{-b\cdot{D}}+f\cdot{e}^{-b\cdot(D+D*)}]$$

where *f* represents the fraction of the fast diffusion component, D corresponds to the true ADC, and D∗ represents the pseudo ADC associated with microcirculation.

**Fig. 2:**
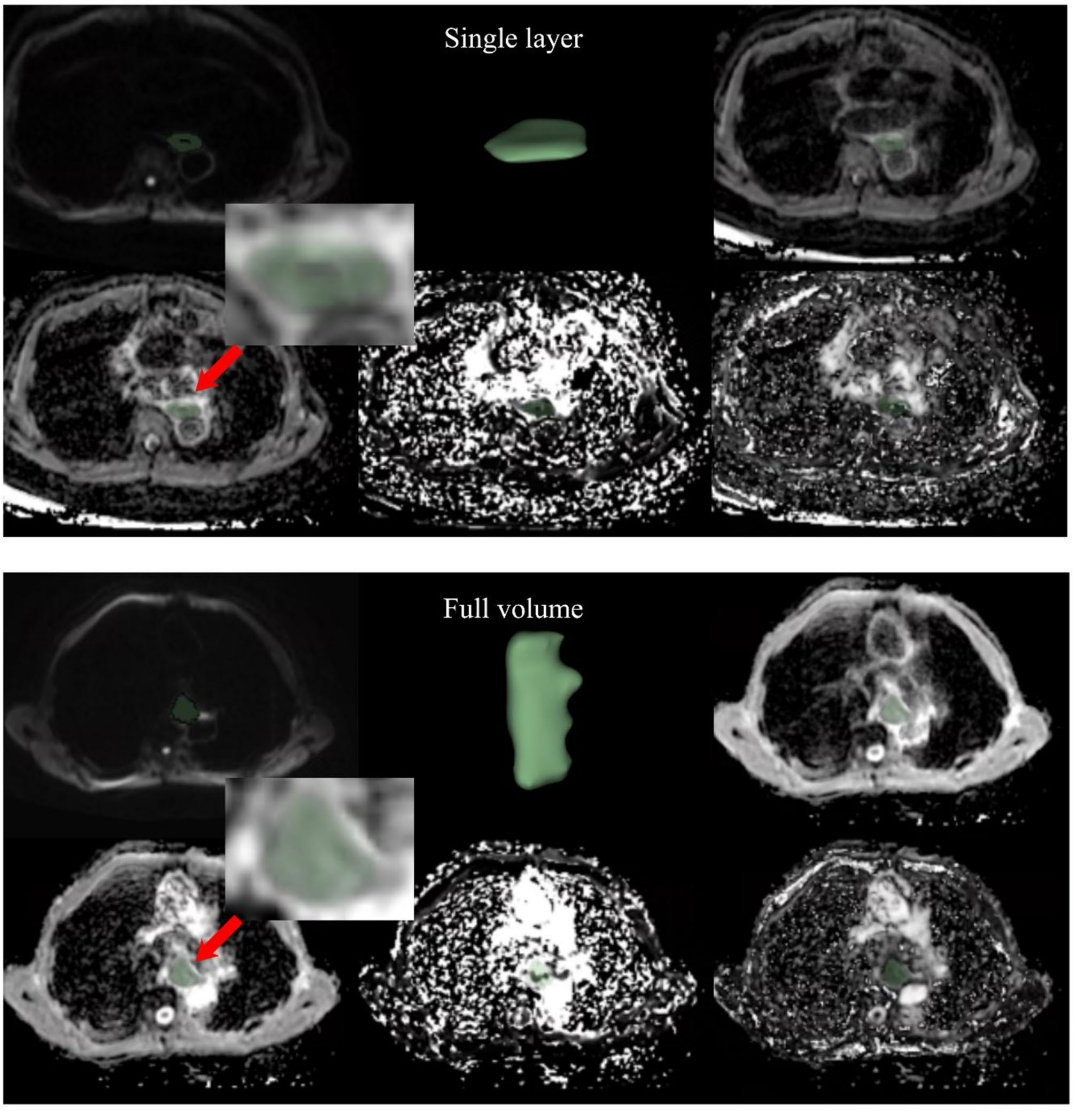
Illustration of maximum-diameter slice and whole volume delineation. The top row from left to right shows: b400 DWI image, 3D display image after delineation, and ADC image; the bottom row from left to right shows: D image, D* image, and f image

For each patient, the two radiologists independently delineated the region of interest (ROI) on the largest cross-sectional area of the solid tumor components on each MRI image, followed by measurements (Fig. 2). Subsequently, the entire tumor volume was delineated and measured. The delineation process ensured coverage of as much of the tumor’s solid portion as possible while avoiding areas of hemorrhage, calcification, and necrosis visible on ADC, D, D∗, and *f* maps. After computing the mean, standard deviation (SD), maximum, and minimum values of ADC, D, D*, and f, the intraclass correlation coefficient (ICC) between the two raters was calculated using the ICC (3, k) model to assess interobserver reliability.

In addition, the two radiologists assessed tumor T staging on high-resolution T2WI, focusing primarily on involvement of the esophageal adventitia. Lymph node status was evaluated by measuring the short-axis diameter of mediastinal lymph nodes, with nodes larger than 10 mm considered indicative of potential metastasis.

### Pathologic examination

Based on the 8th edition of the American Joint Committee on Cancer (AJCC) TNM cancer categories for EC, the T staging of surgically resected specimens was assessed by a pathologist with 15 years of experience, who was blinded to the IVIM data. Patients were classified into two groups based on whether the tumor had infiltrated the esophageal adventitia: T1-T2 and T3-T4a. Additionally, patients were categorized into N- and N + groups according to the presence or absence of lymph node metastasis.

### Statistical analysis

The study data were analyzed using R software (version 4.4.2). Continuous variables were presented as mean ± standard deviation ($\bar{\text{x}}$ ± s) if they conformed to a normal distribution (determined by the Shapiro-Wilk test), or as median (interquartile range) if they did not. T-tests or Mann-Whitney U tests were employed to assess differences between groups for T1-T2 vs. T3-T4a and N- vs. N + classifications. Parameters with an ICC exceeding 0.7 were incorporated into a stepwise regression analysis. Variance inflation factor (VIF) values were evaluated before and after stepwise regression to ensure that the final model was not influenced by multicollinearity. A nomogram was then constructed to visualize the predictive utility of each parameter for T and N staging. Finally, the average receiver operating characteristic (ROC) curve derived from 5-fold cross-validation was generated based on the logistic regression model to evaluate the diagnostic performance of each parameter in determining the T and N stages of ESCC patients. Additionally, out-of-fold predictions from the best-performing model were collected for each patient (i.e., each patient was predicted exactly once in the fold in which they served as the validation set) and directly compared with the per-patient visual assessments made by radiologists. The maximum accuracy achievable by model-assisted human readings was calculated based on the optimal probability threshold. McNemar’s test was performed to evaluate the statistical significance of differences between the readers.

## Results

### Study population

A total of 80 patients were enrolled in this study. The median age of the cohort was 70 years (Q1: 66, Q3: 74). Among the participants, 61 were male and 19 were female. Based on tumor staging, 45 patients (56.3%) were classified as T1-2 stage, while 35 patients (43.7%) were classified as T3-T4a stage (Online Resource Table S1).

### Inter-observer consensus of IVIM parameters and ADC

The volume measurements obtained using both delineation methods showed good consistency. The ICC for maximum-diameter slice delineation was 0.97 (95% CI: 0.96–0.98), while for whole-volume delineation, the ICC was 0.99 (95% CI: 0.99–0.99). For ADC, the consistency of the mean and standard deviation (std) for whole-volume delineation was significantly better, with ICC values both being 0.73. However, the maximum (max) and minimum (min) values showed better consistency in maximum-diameter slice measurement, with ICCs of 0.84 and 0.88, respectively.

In the comparison of IVIM parameters D, D* and *f*, whole-volume delineation consistently demonstrated better inter-observer agreement, especially for mean and std, with ICC values greater than 0.85 for all of them, as detailed in Table 1.

**Table 1:** ICC analysis of parameters at the largest Cross-Sectional area and whole volume

Measurement method	Maximum-diameter slice				Full volume			
Rater	Observer 1	Observer 2	ICC	CI 95%	Observer 1	Observer 2	ICC	CI 95%
Volume/(mm^3^)	1326.4(793.5, 1800.1)	1776.4(1367.8, 2755.4)	0.97	0.96–0.98	6087.30(2711.90,12367.00)	6099.10(2898.60,12618.60)	0.99	0.99–0.99
ADC								
mean/(um^2^/s)	1806.90(1547.10, 2016.60)	1829.00(1653.60, 2063.10)	0.14	-0.34-0.45	1838.40(1644.20,2002.10)	1784.10(1593.80,2036.80)	0.73	0.58–0.83
std/(um^2^/s)	256.10(202.20, 307.30)	257.80(212.10,319.50)	0.44	0.12–0.64	249.90(203.30,300.90)	234.00(198.60,304.70)	0.73	0.59–0.83
max/(um^2^/s)	2398.60(2234.70,2564.30)	2420.40(2275.10,2606.20)	0.84	0.75–0.90	2662.20(2503.40, 2821.60)	2524.10(2351.30,2738.10)	0.41	0.08–0.62
min/(um^2^/s)	1142.66 ± 469.92	1180.02 ± 460.10	0.88	0.81–0.92	948.00(649.10,1198.30)	939.90(663.70,1290.90)	0.22	-0.22-0.50
D								
mean/(um^2^/s)	1960.50 ± 453.30	1970.30 ± 437.70	0.97	0.95–0.98	1972.90(1778.40,2233.20)	1949.50(1736.30,2220.90)	0.92	0.88–0.95
std/(um^2^/s)	319.00(257.20, 379.10)	326.40(254.30,400.30)	0.91	0.86–0.94	325.30(258.40,357.50)	301.60(257.80,350.80)	0.85	0.78–0.91
max/(um^2^/s)	2731.60(2481.40, 3067.90)	2813.40(2521.40, 3058.60)	0.02	-0.53-0.37	3103.40(2870.30,3311.90)	3025.50(2759.00,3324.50)	0.72	0.56–0.82
min/(um^2^/s)	1247.10(969.60, 1564.50)	1264.40(973.30, 1484.40)	0.90	0.84–0.94	979.30(573.70,1262.00)	861.50(546.40,1269.00)	0.56	0.31–0.72
*f*								
mean	0.28(0.23, 0.34)	0.27(0.21, 0.33)	0.93	0.88–0.95	0.30(0.30,0.30)	0.30(0.30,0.30)	0.93	0.90–0.96
std	0.10(0.10, 0.20)	0.10(0.10, 0.20)	0.86	0.78–0.91	0.16 ± 0.05	0.16 ± 0.05	0.88	0.82–0.93
max	0.80(0.60, 1.00)	1.00(0.60, 1.00)	0.00	-0.56-0.36	1.00(1.00, 1.00)	1.00(1.00, 1.00)	0.89	0.83–0.93
min	0.00(0.00,0.10)	0.00(0.00,0.10)	0.00	-0.56-0.36	0.00(0.00,0.00)	0.00(0.00,0.00)	0.88	0.81–0.92
D*								
mean/(um^2^/s)	36087.60(25706.60, 54368.20)	33115.10(24376.70, 48433.00)	0.81	0.71–0.88	38367.70(32050.90,46875.60)	35901.00(29302.70,44905.10)	0.90	0.84–0.93
std/(um^2^/s)	28729.72 ± 10973.63	28828.03 ± 10150.94	0.77	0.65–0.85	28804.51 ± 6910.66	28356.96 ± 7139.25	0.91	0.86–0.94
max/(um^2^/s)	100000.00(100000.00,100000.00)	100000.00(100000.00,100000.00)	0.07	-0.45-0.40	100000.00(100000.00,100000.00)	100000.00(100000.00,100000.00)	0.34	-0.03-0.58
min/(um^2^/s)	1198.00(160.30, 2795.40)	1084.50(616.00,2212.80)	0.57	0.33–0.72	0(0,1034.70)	19.80(0,952.70)	0.89	0.82–0.93

ICC: Intra-class correlation coefficients; std: Standard deviation; max: Maximum; min: Minimum

Due to the slice thickness of MR images, the measurements obtained, even from a single slice, represent volumetric data rather than purely area data

### Comparison of ADC and IVIM parameters between group T1 + T2 and group T3 + T4a

The effectiveness of parameters measured at maximum-diameter slice measurement and full volume in predicting T staging was not entirely consistent (Online Resource Table S1). After excluding parameters with an intraclass ICC < 0.7, significant differences between groups were observed for the following parameters measured at maximum-diameter slice: volume, D_mean, D_min, and *f*_mean. However, not all of these parameters were able to effectively distinguish T stages. Stepwise regression analysis identified volume, ADC_max, ADC_min, and D_min as the most relevant parameters, with ADC_max (*p* = 0.029) and D_min (*p* = 0.013) considered independent risk factors for T staging.

For full volume measurements, parameters showing significant inter-group differences included volume, D_mean, D_min, *f*_mean, *f*_max and *f*_min. After stepwise regression, ADC_std, D_min, *f* _mean, and *f*_max were found to be able to differentiate patients with T1-2 versus T3-T4a stages. Among these, ADC_std (*p* = 0.032) was considered an independent risk factor (Table 2). The VIF values of IVIM and ADC parameters included in the models were all less than 5.

### Comparison of ADC and IVIM parameters between group N- and group N+

The prediction of N staging differed from that of T staging. No significant differences were observed between patients with or without lymph node metastasis for parameters measured at maximum-diameter slice measurement (Online Resource Table S2). Stepwise regression analysis showed that only D_mean could reliably differentiate the presence or absence of lymph node metastasis.

Although parameters measured at full volume did not show clear differences between groups, stepwise regression revealed that D_max, *f*_mean, *f*_min, D*_std, and D*_min were capable of distinguishing patients with lymph node metastasis (Table 2). The VIF values of IVIM and ADC parameters included in the models were all less than 5.

**Table 2:**
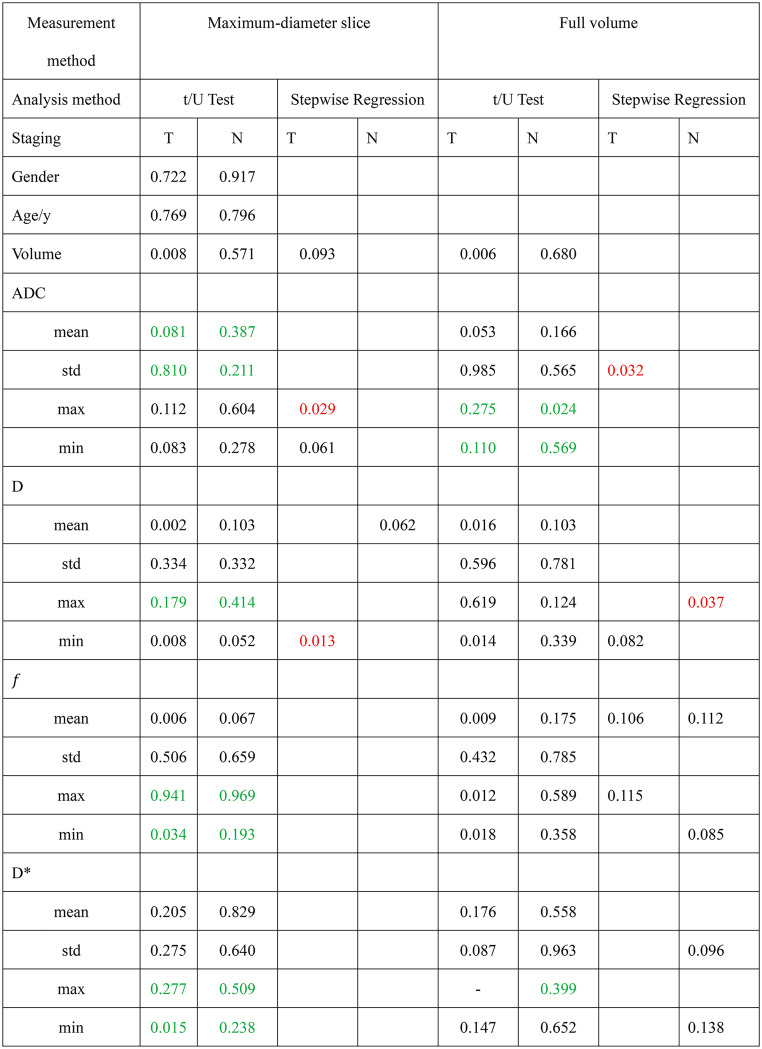
Intragroup difference testing and multivariate analysis results after stepwise regression

Continuous variables were analyzed using an independent samples t-test if normally distributed; otherwise, the Mann-Whitney U test was employed. Parameters with an intraclass ICC < 0.7 (denoted by green numbers) were excluded from subsequent analyses to address poor inter-observer consistency. Stepwise regression was performed to screen variables for inclusion in a multivariate logistic regression model assessing associations with T and N staging. Red numbers indicate parameters identified as significantly related to staging in the final multivariate analysis (*p* < 0.05), while “-” signifies identical values between groups, precluding intragroup difference testing

### Performance of logistic regression models in predicting T staging and N staging

**Fig. 3:**
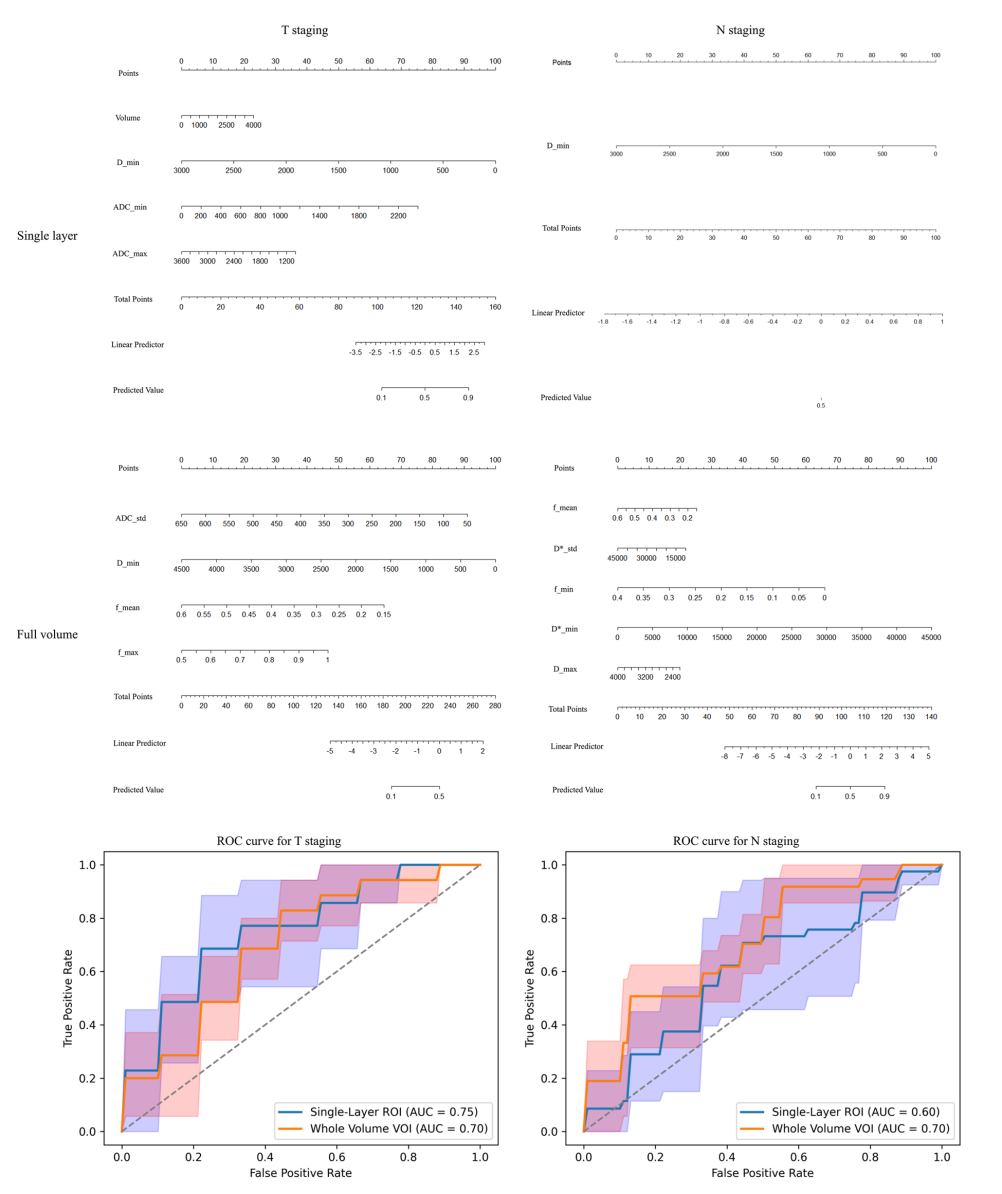
Nomogram and ROC curves for predicting T3-T4a stage and lymph node metastasis using different measurement approaches. Each predictor is assigned a corresponding score based on its value (top scales for individual variables). The total score (sum of individual scores, middle scale) is mapped to the probability of T3-T4a stage (T staging, left) and lymph node metastasis (N staging, right). Inset ROC curves (left panel: T staging; right panel: N staging) depict the average diagnostic performance of the logistic regression model across 5-fold cross-validation, with the shaded area representing the 95% confidence interval (CI) of the AUC

The nomogram (Fig. 3) visually illustrates the relative contributions of the selected predictors within the logistic regression model for T/N staging. Meanwhile, the ROC curve analysis demonstrates the predictive performance of the models developed via stepwise regression. For T staging, the maximum-diameter slice measurement achieved a higher AUC (0.75 vs. 0.70) and specificity (0.778 vs. 0.667) compared to full-volume measurements. In contrast, for predicting lymph node metastasis, the full-volume method demonstrated superior AUC (0.70 vs. 0.60) and sensitivity (0.625 vs. 0.354), despite a slight decrease in specificity (0.631 vs. 0.772) (Table 3).

**Table 3:**
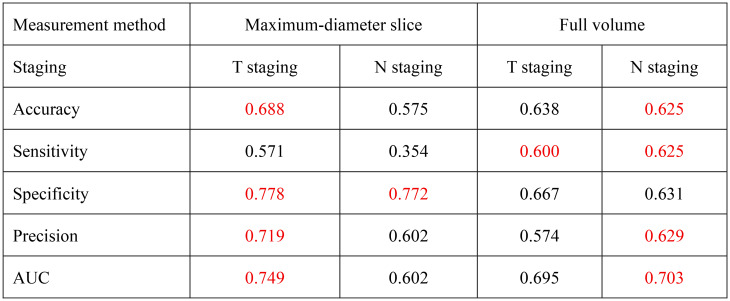
Performance metrics comparison for T and N staging

The table summarizes the average classification performance of maximum-diameter slice measurement and full-volume approaches for T and N staging based on 5-fold cross-validation. Metrics include Accuracy, Sensitivity, Specificity, Precision, and AUC, with superior values highlighted in red

### Model-assisted radiologist performance

The out-of-fold predictions (generated from the rotating validation folds in 5-fold cross-validation) were compared on a case-by-case basis with the independent visual assessments of two radiologists (Observer 1 and Observer 2), with each observer’s evaluation detailed in Supplementary Tables S3 and S4. Under ideal conditions, model-assisted interpretation substantially improved sensitivity for both T and N staging (T staging: from 0.657/0.743 to 0.971; N staging: from 0.378/0.405 to 0.757), while maintaining high accuracy and specificity (Table 4). McNemar’s test P-values for T staging were 0.332 and 0.789, and for N staging, 0.002 and 0.010.

**Table 4:** Diagnostic performance metrics for T and N staging: radiologist assessment with or without model assistance

	Model 1 Threshold = 0.368	Model 2 Threshold = 0.456	Observer 1	Observer 2	Observer 1 (Model-assisted)	Observer 2 (Model-assisted)
T staing						
Accuracy	0.700	-	0.688	0.700	0.900	0.875
Sensitivity	0.829	-	0.657	0.743	0.971	0.971
Specificity	0.600	-	0.711	0.667	0.844	0.800
Precision	0.617	-	0.639	0.634	0.829	0.791
N staging						
Accuracy	-	0.625	0.663	0.663	0.850	0.850
Sensitivity	-	0.676	0.378	0.405	0.757	0.757
Specificity	-	0.581	0.907	0.884	0.930	0.930
Precision	-	0.581	0.778	0.750	0.903	0.903

Model 1 refers to the out-of-fold results of the model trained and validated using maximum-diameter slice measurements, while Model 2 refers to the out-of-fold results of the model trained and validated using full-volume measurements

## Discussion

This study evaluated the diagnostic performance of ADC and IVIM parameters measured using different methods in assessing T-staging and N-staging in EC patients. The results indicated that the diagnostic efficacy of ADC and IVIM parameters varies depending on the measurement approach. Specifically, parameters derived from the maximum-diameter slice measurement provide superior accuracy in determining T-staging (AUC: 0.75). Whole-tumor volume-based parameters exhibited higher effectiveness in assessing lymph node metastasis (AUC: 0.70).

Preoperative T-staging and lymph node metastasis evaluation are critical for surgical decision-making and the selection of adjuvant treatment strategies [17, 18]. Recent advances in MRI have facilitated its growing application in EC research [19]. IVIM and ADC imaging, which quantify water molecule diffusion and microcirculation perfusion, provide comprehensive insights into tumor cell density, microvascular proliferation, and the tumor microenvironment. These techniques have increasingly been validated for their utility in tumor staging [6].

As EC progresses, cancer-associated fibroblasts remodel the extracellular matrix (ECM) by secreting excessive amounts of ECM proteins such as collagen and fibronectin, forming dense collagen fiber networks. This leads to ECM thickening and stiffening [20], further restricting water molecule diffusion. ADC reflects the average diffusion capacity of water molecules in tissues, while IVIM separates diffusion from perfusion effects, with the true ADC (D) representing pure diffusion. Both parameters are closely associated with tumor cell density and extracellular space characteristics [21], supporting their potential role in delineating tumor boundaries. Previous studies by Ryoya M and Tao S et al. demonstrated that ADC and D values decrease with higher T-stages [22, 23]. Similarly, our findings confirmed that ADC and D values in the T3-T4a group were significantly lower than those in the T1-T2 group, with ADCmax, measured at the maximum-diameter slice, identified as an independent risk factor. A comparison of whole-tumor volume and maximum-diameter slice measurements revealed that although similar trends were observed in whole-volume measurements, stepwise regression analysis indicated inferior performance in distinguishing EC invasion of the outer membrane. This discrepancy might arise because the maximum-diameter slice typically represents the most expansive tumor region, where outer membrane invasion is more likely, whereas whole-tumor volume measurements incorporate non-invasive regions, potentially introducing diagnostic noise.

Additionally, in lymph nodes infiltrated by malignant cells, not only does the D value decrease, but D* and *f* values, which reflect microvascular permeability and perfusion, also decline [24]. These changes were validated in EC primary lesions. Studies by Tao S et al. demonstrated that D, *f*, and ADC values were significantly lower in the lymph node metastasis group than in the non-metastatic group, while D* exhibited a similar trend without statistical significance (*P* >0.05) [23]. Our findings align with these results, with further analysis suggesting that whole-tumor volume-based parameters are more effective in distinguishing lymph node metastasis. Among these, Dmax was identified as an independent risk factor, while D* and *f* parameters also served as effective discriminative markers. This may be attributed to whole-volume measurements better capturing tumor microenvironment heterogeneity and angiogenesis.

By quantitatively analyzing EC lesions using IVIM and ADC parameters, subjective bias in imaging interpretation can be minimized, providing more objective reference data. These parameters show promise as significant imaging biomarkers for preoperative staging, differentiation, and potentially predicting treatment response in EC [25, 26].

However, this study has several limitations. First, the exclusion of patients who had received neoadjuvant therapy may have resulted in a sample with limited representativeness, as many T3–T4 and node-positive cases were systematically excluded, potentially leading to underestimation or overestimation of ADC and IVIM parameters in these patients.Second, manual delineation of esophageal carcinoma ROIs may introduce subjectivity, underscoring the need for standardized automated volumetric analysis to enhance measurement reproducibility. Lastly, the single-center design and limited sample size may compromise the generalizability of findings, necessitating validation through multicenter studies with larger cohorts.

## Conclusion

MRI-derived IVIM and ADC parameters provide non-invasive biomarkers for ESCC staging and may assist in preoperative decision-making, while different ROI delineation strategies could foster new research advances and offer stronger evidence for clinical translation.

## Supplementary Information

Below is the link to the electronic supplementary material.

Supplementary Material 1

## Data Availability

The datasets generated during and/or analysed during the current study are available from the corresponding author on reasonable request.
