# Supplementary material for: Preoperative esophageal cancer staging assessment based on intravoxel incoherent motion and apparent diffusion coefficient: a comparative study of maximum-diameter slice region of interest and whole volume of interest analysis
Source: BMC Med Imaging. 2025 Nov 4;25:444. doi: 10.1186/s12880-025-01973-x (PMC12584429; doi:10.1186/s12880-025-01973-x)
Supplement: Supplementary file 1 — Supplementary Material 1 [file 12880_2025_1973_MOESM1_ESM.docx]

Table S1: Analysis of Differences Between T Staging Groups Using Different Measurement Approaches

| Measurement method | Single layer | | | Full volume | | |
| --- | --- | --- | --- | --- | --- | --- |
| T staging | T1-2 (n = 45) | T3-4a (n = 35) | p | T1-2 (n = 45) | T3-4a (n = 35) | p |
| Gender |  |  |  |  |  |  |
| male | 35(78.8%) | 26(74.3%) | 0.921 | - | - | - |
| female | 10(22.2%) | 9(25.7%) |  | - | - |  |
| Age/y | 70±6 | 69±7 | 0.769 |  |  |  |
| Volume/(mm^3^) | 1367.80(823.10, 1788.20) | 1752.70(1421.20,2668.60) | 0.008 | 4.25(1.89,10.53) | 8.98(5.48,13.49) | 0.006 |
| ADC |  |  |  |  |  |  |
| mean/(um^2^/s) | 1885.80(1672.30,2083.30) | 1770.30(1529.40,1947.80) | 0.081 | 1868.10(1633.90,5015.30) | 1745.40(1594.70,1938.10) | 0.053 |
| std/(um^2^/s) | 260.10(183.80,330.40) | 251.80(217.50,298.00) | 0.810 | 243.00(192.40,296.70) | 234.40(208.70,297.60) | 0.985 |
| max/(um^2^/s) | 2461.60(2293.90,2680.30) | 2361.30(2253.20,2472.90) | 0.112 | 2618.80(2465.50,2821.20) | 2558.10(2368.30,2734.70) | 0.275 |
| min/(um^2^/s) | 1235.42±449.59 | 1066.09±410.68 | 0.083 | 962.90(731.50,1337.50) | 856.60(685.70,1023.60) | 0.110 |
| D |  |  |  |  |  |  |
| mean/(um^2^/s) | 2096.62±408.54 | 1796.76±424.19 | 0.002 | 2098.50(1789.70, 2287.70) | 1873.00(1671.00, 2143.90) | 0.016 |
| std/(um^2^/s) | 323.70(220.850,373.50) | 326.40(279.60,397.50) | 0.334 | 311.50(246.90, 356.40) | 307.70(283.50, 347.60) | 0.596 |
| max/(um^2^/s) | 2837.50(2469.60,3171.90) | 2715.50(2559.40,2909.70) | 0.179 | 3061.55±365.09 | 3022.90±325.90 | 0.619 |
| min/(um^2^/s) | 1349.70(1069.50,1708.00) | 1050.90(777.00,1456.30) | 0.008 | 1078.70(573.50, 1436.30) | 815.80(454.00, 1027.90) | 0.014 |
| 𝑓 |  |  |  |  |  |  |
| mean | 0.30(0.24,0.37) | 0.30(0.19,0.31) | 0.006 | 0.30(0.27, 0.37) | 0.30(0.24, 0.31) | 0.009 |
| std | 0.10(0.10,0.21) | 0.10(0.10,0.20) | 0.506 | 0.15±0.05 | 0.16±0.05 | 0.432 |
| max | 0.80(0.62,1.00) | 0.90(0.59,1.00) | 0.941 | 1.00(0.80,1.00) | 1.00(1.00,1.00) | 0.012 |
| min | 0.00(0.00,0.14) | 0.00(0.00,0.05) | 0.034 | 0.00(0.00,0.04) | 0.00(0.00,0.00) | 0.018 |
| D* |  |  |  |  |  |  |
| mean/(um^2^/s) | 41332.90(27803.30,49825.10) | 32523.80(23280.40,42566.20) | 0.205 | 40458.60(33057.30,47852.20) | 36260.90(31089.40,40260.90) | 0.176 |
| std/(um^2^/s) | 27735.47±9238.06 | 30120.39±9895.14 | 0.275 | 27456.12±6863.13 | 30026.67±6364.81 | 0.087 |
| max/(um^2^/s) | ~ | ~ | ~ | ~ | ~ | ~ |
| min/(um^2^/s) | 1851.60(598.90,5495.90) | 803.60(312.90,1452.80) | 0.015 | 195.90(0.00,1195.70) | 39.50(0.00,501.60) | 0.147 |

"~" indicates that the values in the two groups are identical, and the P-value cannot be calculated.

Table S2: Analysis of Differences Between N Staging Groups Using Different Measurement Approaches

| Measurement method | Single layer | | | Full volume | | |
| --- | --- | --- | --- | --- | --- | --- |
| N staging | N- (n =43) | N+ (n =37) | p | N- (n =43) | N+ (n = 37) | p |
| Gender |  |  |  |  |  |  |
| male | 33(76.7%) | 28(75.7%) | 0.917 | - | - | - |
| female | 10(23.3%) | 9(24.3%) |  | - | - |  |
| Age/y | 69±7 | 70±7 | 0.796 | 69±7 | 70±7 | 0.796 |
| Volume/(mm^3^) | 1533.70(1039.20, 2137.60) | 1539.60(1154.70,2143.60) | 0.571 | 5501.0(2190.00,13184.10) | 6756.4(3961.4,11783.6) | 0.680 |
| ADC |  |  |  |  |  |  |
| mean/(um^2^/s) | 1811.40(1642.10,2103.70) | 1787.50(1617.30,1928.70) | 0.387 | 1868.10(1573.90,2041.70) | 1762.50(1625.60,1930.40) | 0.166 |
| std/(um^2^/s) | 246.90(192.30,322.10) | 271.30(223.50,313.10) | 0.211 | 243.30(204.50,291.60) | 227.80(196.60,301.70) | 0.565 |
| max/(um^2^/s) | 2397.30(2253.60,2669.90) | 2399.70(2275.10,2546.90) | 0.604 | 2657.60(2466.00,2918.40) | 2523.40(2382.10,2680.70) | 0.024 |
| min/(um^2^/s) | 1210.18±478.93 | 1104.58±385.05 | 0.280 | 907.30(713.00,1321.10) | 859.30(721.50,1131.40) | 0.569 |
| D |  |  |  |  |  |  |
| mean/(um^2^/s) | 1964.90(1804.00, 2358.40) | 1885.00(1666.90, 2067.50) | 0.103 | 2098.50(1694.90, 2293.20) | 1873.00(1771.90, 2126.80) | 0.103 |
| std/(um^2^/s) | 308.30(220.80,371.70) | 333.20(272.20,395.60) | 0.332 | 316.90(272.10, 355.00) | 301.80(275.10, 351.20) | 0.781 |
| max/(um^2^/s) | 2835.00(2533.50,3142.30) | 2734.70(2577.50,2962.00) | 0.414 | 3099.96±348.58 | 2980.34±338.19 | 0.124 |
| min/(um^2^/s) | 1361.52±574.76 | 1136.33±442.63 | 0.052 | 1007.00(524.90, 1402.30) | 827.00(507.60, 1155.20) | 0.339 |
| 𝑓 |  |  |  |  |  |  |
| mean | 0.30(0.24,0.38) | 0.30(0.21,0.32) | 0.067 | 0.30(0.26,0.35) | 0.30(0.25,0.32) | 0.175 |
| std | 0.10(0.10,0.21) | 0.10(0.10,0.19) | 0.659 | 0.16±0.05 | 0.16±0.05 | 0.785 |
| max | 0.80(0.60,1.00) | 0.80(0.60,1.00) | 0.969 | 1.00(0.94,1.00) | 1.00(1.00,1.00) | 0.589 |
| min | 0.00(0.00,0.13) | 0.00(0.00,0.06) | 0.193 | 0.00(0.00,0.00) | 0.00(0.00,0.00) | 0.358 |
| D* |  |  |  |  |  |  |
| mean/(um^2^/s) | 38732.17±16108.75 | 39612.62±19595.28 | 0.829 | 38233.10(33180.00,45127.80) | 36539.60(30768.20,45459.90.40) | 0.558 |
| std/(um^2^/s) | 28307.74±9029.62 | 29326.41±10207.59 | 0.640 | 28613.55±6394.57 | 28542.59±7192.72 | 0.963 |
| max/(um^2^/s) | 1e+05(1e+05,1e+05) | 1e+05(1e+05,1e+05) | 0.509 | 1e+05(1e+05,1e+05) | 1e+05(1e+05,1e+05) | 0.399 |
| min/(um^2^/s) | 1817.10(476.70,4576.40) | 889.20(514.00,1697.40) | 0.238 | 39.5(0.00,951.50) | 223.40(0.00,799.10) | 0.652 |

Table S3 Case-by-case comparison of T staging between observers and model predictions

| list | T staging | Observer 1 | Observer 2 | Model Predict |
| --- | --- | --- | --- | --- |
| 1 | 0 | 1 | 1 | 0.26 |
| 2 | 1 | 1 | 1 | 0.58 |
| 3 | 1 | 1 | 1 | 0.66 |
| 4 | 1 | 0 | 0 | 0.83 |
| 5 | 1 | 1 | 1 | 0.78 |
| 6 | 1 | 1 | 1 | 0.44 |
| 7 | 0 | 0 | 0 | 0.37 |
| 8 | 1 | 1 | 1 | 0.40 |
| 9 | 0 | 0 | 1 | 0.44 |
| 10 | 1 | 0 | 0 | 0.51 |
| 11 | 0 | 0 | 0 | 0.20 |
| 12 | 0 | 1 | 0 | 0.70 |
| 13 | 1 | 1 | 1 | 0.54 |
| 14 | 0 | 1 | 1 | 0.37 |
| 15 | 0 | 0 | 0 | 0.16 |
| 16 | 1 | 0 | 1 | 0.43 |
| 17 | 1 | 1 | 1 | 0.37 |
| 18 | 1 | 1 | 0 | 0.39 |
| 19 | 0 | 0 | 0 | 0.28 |
| 20 | 0 | 0 | 0 | 0.18 |
| 21 | 0 | 0 | 0 | 0.17 |
| 22 | 0 | 0 | 0 | 0.29 |
| 23 | 0 | 0 | 0 | 0.13 |
| 24 | 0 | 1 | 1 | 0.38 |
| 25 | 0 | 0 | 0 | 0.15 |
| 26 | 1 | 1 | 1 | 0.66 |
| 27 | 1 | 0 | 0 | 0.58 |
| 28 | 1 | 1 | 1 | 0.66 |
| 29 | 1 | 0 | 1 | 0.58 |
| 30 | 1 | 1 | 1 | 0.58 |
| 31 | 1 | 1 | 1 | 0.33 |
| 32 | 1 | 1 | 1 | 0.39 |
| 33 | 0 | 0 | 1 | 0.43 |
| 34 | 1 | 1 | 1 | 0.30 |
| 35 | 0 | 0 | 0 | 0.11 |
| 36 | 1 | 1 | 1 | 0.51 |
| 37 | 0 | 0 | 0 | 0.05 |
| 38 | 1 | 0 | 0 | 0.62 |
| 39 | 0 | 0 | 0 | 0.55 |
| 40 | 0 | 1 | 1 | 0.80 |
| 41 | 1 | 0 | 1 | 0.86 |
| 42 | 1 | 0 | 1 | 0.81 |
| 43 | 0 | 0 | 0 | 0.33 |
| 44 | 1 | 0 | 1 | 0.46 |
| 45 | 1 | 0 | 0 | 0.35 |
| 46 | 1 | 0 | 0 | 0.49 |
| 47 | 1 | 1 | 0 | 0.57 |
| 48 | 1 | 0 | 1 | 0.56 |
| 49 | 0 | 1 | 1 | 0.89 |
| 50 | 0 | 0 | 0 | 0.49 |
| 51 | 0 | 1 | 1 | 0.34 |
| 52 | 1 | 1 | 1 | 0.26 |
| 53 | 0 | 0 | 0 | 0.27 |
| 54 | 1 | 1 | 1 | 0.78 |
| 55 | 0 | 0 | 0 | 0.36 |
| 56 | 1 | 1 | 1 | 0.73 |
| 57 | 0 | 1 | 1 | 0.56 |
| 58 | 0 | 1 | 1 | 0.40 |
| 59 | 1 | 1 | 1 | 0.34 |
| 60 | 0 | 1 | 0 | 0.56 |
| 61 | 0 | 0 | 0 | 0.65 |
| 62 | 0 | 0 | 0 | 0.24 |
| 63 | 0 | 1 | 1 | 0.34 |
| 64 | 0 | 0 | 0 | 0.44 |
| 65 | 0 | 0 | 0 | 0.28 |
| 66 | 0 | 0 | 0 | 0.31 |
| 67 | 1 | 1 | 1 | 0.22 |
| 68 | 0 | 0 | 1 | 0.63 |
| 69 | 0 | 0 | 0 | 0.49 |
| 70 | 0 | 1 | 1 | 0.31 |
| 71 | 1 | 1 | 1 | 0.74 |
| 72 | 0 | 0 | 1 | 0.11 |
| 73 | 0 | 0 | 0 | 0.50 |
| 74 | 0 | 1 | 0 | 0.34 |
| 75 | 0 | 0 | 1 | 0.44 |
| 76 | 0 | 0 | 0 | 0.30 |
| 77 | 1 | 1 | 0 | 0.46 |
| 78 | 0 | 0 | 0 | 0.26 |
| 79 | 0 | 0 | 0 | 0.90 |
| 80 | 0 | 0 | 0 | 0.30 |

T1 indicates that the esophageal tumor has invaded the adventitia, whereas T0 indicates no invasion. Observer 1 and Observer 2 represent the independent assessments of two radiologists. "Model Predict" shows the probability of adventitial invasion predicted by the model.

Table S4 Case-by-case comparison of N staging between observers and model predictions

| list | N staging | Observer 1 | Observer 2 | Model Predict |
| --- | --- | --- | --- | --- |
| 1 | 0 | 0 | 0 | 0.44 |
| 2 | 1 | 1 | 1 | 0.41 |
| 3 | 0 | 0 | 0 | 0.48 |
| 4 | 0 | 0 | 0 | 0.56 |
| 5 | 1 | 0 | 0 | 0.45 |
| 6 | 1 | 0 | 0 | 0.37 |
| 7 | 0 | 0 | 0 | 0.52 |
| 8 | 1 | 1 | 1 | 0.46 |
| 9 | 0 | 0 | 0 | 0.49 |
| 10 | 0 | 0 | 0 | 0.54 |
| 11 | 0 | 0 | 0 | 0.42 |
| 12 | 0 | 0 | 0 | 0.45 |
| 13 | 1 | 1 | 1 | 0.51 |
| 14 | 0 | 1 | 1 | 0.50 |
| 15 | 0 | 0 | 0 | 0.43 |
| 16 | 1 | 0 | 0 | 0.49 |
| 17 | 0 | 0 | 0 | 0.49 |
| 18 | 0 | 0 | 0 | 0.45 |
| 19 | 0 | 0 | 0 | 0.44 |
| 20 | 1 | 1 | 1 | 0.44 |
| 21 | 1 | 0 | 0 | 0.19 |
| 22 | 1 | 0 | 0 | 0.47 |
| 23 | 0 | 0 | 0 | 0.31 |
| 24 | 1 | 1 | 1 | 0.49 |
| 25 | 0 | 1 | 1 | 0.36 |
| 26 | 1 | 0 | 0 | 0.47 |
| 27 | 1 | 0 | 0 | 0.56 |
| 28 | 0 | 0 | 0 | 0.68 |
| 29 | 0 | 0 | 0 | 0.49 |
| 30 | 1 | 1 | 1 | 0.56 |
| 31 | 1 | 0 | 0 | 0.30 |
| 32 | 1 | 1 | 1 | 0.49 |
| 33 | 0 | 0 | 0 | 0.37 |
| 34 | 1 | 0 | 0 | 0.47 |
| 35 | 0 | 0 | 0 | 0.28 |
| 36 | 1 | 1 | 1 | 0.54 |
| 37 | 0 | 0 | 0 | 0.22 |
| 38 | 0 | 0 | 0 | 0.65 |
| 39 | 0 | 0 | 0 | 0.40 |
| 40 | 1 | 1 | 1 | 0.40 |
| 41 | 1 | 0 | 0 | 0.72 |
| 42 | 1 | 0 | 0 | 0.38 |
| 43 | 0 | 0 | 0 | 0.43 |
| 44 | 1 | 0 | 0 | 0.42 |
| 45 | 0 | 0 | 0 | 0.46 |
| 46 | 1 | 1 | 1 | 0.47 |
| 47 | 1 | 0 | 0 | 0.61 |
| 48 | 1 | 0 | 1 | 0.55 |
| 49 | 0 | 1 | 1 | 0.60 |
| 50 | 0 | 0 | 0 | 0.45 |
| 51 | 0 | 0 | 0 | 0.39 |
| 52 | 1 | 0 | 0 | 0.39 |
| 53 | 0 | 0 | 0 | 0.33 |
| 54 | 1 | 1 | 1 | 0.53 |
| 55 | 1 | 0 | 0 | 0.51 |
| 56 | 1 | 0 | 0 | 0.56 |
| 57 | 1 | 1 | 1 | 0.56 |
| 58 | 0 | 0 | 0 | 0.36 |
| 59 | 1 | 1 | 1 | 0.47 |
| 60 | 0 | 0 | 0 | 0.51 |
| 61 | 0 | 0 | 0 | 0.61 |
| 62 | 0 | 0 | 0 | 0.38 |
| 63 | 0 | 0 | 0 | 0.45 |
| 64 | 1 | 0 | 0 | 0.43 |
| 65 | 1 | 0 | 0 | 0.41 |
| 66 | 0 | 0 | 0 | 0.41 |
| 67 | 0 | 0 | 0 | 0.39 |
| 68 | 0 | 0 | 0 | 0.49 |
| 69 | 0 | 0 | 0 | 0.53 |
| 70 | 0 | 0 | 0 | 0.44 |
| 71 | 1 | 0 | 0 | 0.47 |
| 72 | 0 | 0 | 1 | 0.40 |
| 73 | 1 | 0 | 0 | 0.55 |
| 74 | 1 | 0 | 0 | 0.49 |
| 75 | 0 | 0 | 0 | 0.47 |
| 76 | 0 | 0 | 0 | 0.38 |
| 77 | 1 | 0 | 0 | 0.55 |
| 78 | 0 | 1 | 1 | 0.49 |
| 79 | 0 | 0 | 0 | 0.60 |
| 80 | 1 | 1 | 1 | 0.46 |

N1 indicates the presence of lymph node metastasis, and N0 indicates no lymph node metastasis. Observer 1 and Observer 2 are independent assessments by two radiologists. "Model Predict" shows the probability of lymph node metastasis predicted by the model.
